# EEG and peripheral markers of viewer ratings: a study of short films

**DOI:** 10.3389/fnins.2023.1148205

**Published:** 2023-06-12

**Authors:** Vladimir Kosonogov, Danila Shelepenkov, Nikita Rudenkiy

**Affiliations:** ^1^Institute for Cognitive Neuroscience, HSE University, Moscow, Russia; ^2^National Research Nuclear University MEPhI, Moscow, Russia

**Keywords:** short films, EEG, heart rate, skin conductance, valence, arousal, *zygomaticus major*, *corrugator supercilii*

## Abstract

**Introduction:**

Cinema is an important part of modern culture, influencing millions of viewers. Research suggested many models for the prediction of film success, one of them being the use of neuroscientific tools. The aim of our study was to find physiological markers of viewer perception and correlate them to short film ratings given by our subjects. Short films are used as a test case for directors and screenwriters and can be created to raise funding for future projects; however, they have not been studied properly with physiological methods.

**Methods:**

We recorded electroencephalography (18 sensors), facial electromyography (*corrugator supercilii* and *zygomaticus major*), photoplethysmography, and skin conductance in 21 participants while watching and evaluating 8 short films (4 dramas and 4 comedies). Also, we used machine learning (CatBoost, SVR) to predict the exact rating of each film (from 1 to 10), based on all physiological indicators. In addition, we classified each film as low or high rated by our subjects (with Logistic Regression, KNN, decision tree, CatBoost, and SVC).

**Results:**

The results showed that ratings did not differ between genres. *Corrugator supercilii* activity (“frowning” muscle) was larger when watching dramas; whereas *zygomaticus major* (“smiling” muscle) activity was larger during the watching of comedies. Of all somatic and vegetative markers, only *zygomaticus major* activity, PNN50, SD1/SD2 (heart rate variability parameters) positively correlated to the film ratings. The EEG engagement indices, beta/(alpha+theta) and beta/alpha correlated positively with the film ratings in the majority of sensors. Arousal (beta_F3_ + beta_F4_)/(alpha_F3_ + alpha_F4_), and valence (alpha_F4_/beta_F4_) - (alpha_F3_/beta_F3_) indices also correlated positively to film ratings. When we attempted to predict exact ratings, MAPE was 0.55. As for the binary classification, logistic regression yielded the best values (area under the ROC curve = 0.62) than other methods (0.51–0.60).

**Discussion:**

Overall, we revealed EEG and peripheral markers, which reflect viewer ratings and can predict them to a certain extent. In general, high film ratings can reflect a fusion of high arousal and different valence, positive valence being more important. These findings broaden our knowledge about the physiological basis of viewer perception and can be potentially used at the stage of film production.

## Introduction

Cinema is an important part of modern popular culture, influencing millions of viewers. In 2021, according to the Theatrical and Home/Mobile Entertainment Market Environment report, 403 films were released in Canada and the US alone, and the combined global theatrical and home/mobile entertainment market reached $99.7 billion (Motion Picture Association, 2022). However, in addition to great budgets, the film industry is characterised by competition and risk. Due to high production costs and marketing budgets, even well-known films may not break even at the box office (income from ticket sales). For example, in 2021, famous films with big marketing budgets like *The Suicide Squad* and *The Last Duel* failed to make enough money at the box office to recoup their budget. Some authors point out that more than 75% of new film releases face a net loss during their run in theatres (Boksem and Smidts, 2015). Because of the risk of losing money, companies in the film industry are turning to various methods of film promotion and prediction of success.

To mitigate such risks, directors, screenwriters, and film studios need to know how audiences would react to films beforehand. Therefore, research suggested many models for the prediction of film success, which is commonly understood as high box office revenue. Typically, such models include information about the actors, locations, budget, release time, polls, etc. (for more detailed information, see Lash and Zhao, 2016; Ahmad et al., 2017). Besides box office receipts, another measure of a film’s success is its rating on websites like *RottenTamatoes*, *Metacritic*, *Kinopoisk,* and *Internet Movie Database (IMDb)*.Scores on websites received from all users and critics are related to viewing revenue and viewing satisfaction (Moon et al., 2010). It was shown that viewer ratings are the most effective predictors of financial income (Dellarocas et al., 2005). Recently, researchers also began to to apply machine-learning algorithms to predict film scores, since this have a very promising significance to the film industry. Thus, Dhir and Raj (2018) found the importance of the number of *Facebook* likes, film genres, the number of critics in reviews in the film score prediction. Latif and Afzal (2016) derived ratings from the number of votes on Oscar awards. In addition, film ratings can be predicted from the facial expression of viewers (Shetty et al., 2021).

This area of knowledge is actively developing and looking for new approaches, one of them being the use of neuroscientific tools and data. This area of research offers a wide range of techniques such as functional magnetic resonance imaging (fMRI; Hasson et al., 2008) and electroencephalography (EEG; Heimann et al., 2014). The most common tool for such studies is the EEG. This is due to the fact that EEG studies are easier to reproduce, EEG is relatively cheap, which is why it can be more applicable to marketing research (Nilashi et al., 2020). Peripheral physiological measures, such as automated facial emotion recognition, heart rate, respiration rate and electrodermal activity (EDA) have been used less often (Aznar et al., 2019). But recent studies show the presence of inter-subject correlation in physiological data during watching movies, such as heart rate (Madsen and Parra, 2022) and galvanic skin response (Palumbo et al., 2017). Research also shows that physiological data such as corrugator and zygomaticus EMG correlate with valence of films (Sato et al., 2020). Overall, these studies show that physiological measures can potentially contribute to predict a film rating and commercial success.

The use of neuroscientific methods has become popular following research showing that they can improve self-reported data (Boksem and Smidts, 2015). Thus, Boksem and Smidts (2015) measured the EEG activity while watching 18 film trailers, and collected behavioural information about liking movies and willingness to pay for tickets. They showed that an important predictor of box office performance was EEG activity around the fronto-central regions in the gamma-band, while the results of the willingness-to-pay poll were not a significant predictor of box office success. This conclusion was confirmed in a following study (Barnett and Cerf, 2017). They measured brain activity of fifty-eight participants with EEG and peripheral data in a commercial theatre while watching film trailers. Then, they calculated the relative levels of neural similarity, which they called cross-brain correlation (CBC). The level of CBC could predict film recall and box office revenue, at the same time the autonomic nervous system data, like EDA, cardiac or respiratory rate, were not associated with recall or box office success (Barnett and Cerf, 2017). However, box office receipts depend on many factors, including distribution related factors (e.g., budget or franchise), brand and star effects (e.g., top actors or directors), and evaluation sources (e.g., critics and audience rating) and region-specific variables (Gaenssle et al., 2018). We focused on the neural correlates of audience rating and user rating prediction, which had rarely been considered in film neuroscience before. However, a recent study has shown that EEG recordings while watching movie trailers can also be successfully used to predict ranks of subjects’ preferences using machine learning methods (Shetty et al., 2021).

The found physiological markers, capable of predicting ratings or commercial success, are usually linked with cognitive states of focused attention, the brain reward network, emotional response, engagement levels, and enjoyment. For example, Christoforou et al. (2017) have found that the gamma-band of EEG while watching trailers significantly predicted box office success on the first weekend and in the following few weeks. They associated gamma-activity while watching, with the trailer or film’s ability to capture the viewer’s attention. Other EEG components related to engagement and enjoyment can be used to predict other aspects of film success such as rating. For example, activity in the beta-band of EEG is usually associated with an individual preference for short-term rewards (Cohen et al., 2007). In this vein, Boksem and Smidts (2015) showed that beta-activity was related to a high viewer preference for films.

We hypothesised that such well-spread EEG markers as engagement indices, beta/(alpha+theta) and beta/alpha (Pope et al., 1995) and valence and arousal indices (Giraldo and Ramirez, 2013) may be other accurate markers for predicting film ratings. Although these indices have been widely used for engagement and workload measurement (Berka et al., 2007; Lelis-Torres et al., 2017; Apicella et al., 2022), to our knowledge, they have not been applied to study film perception and film ratings. The frequency of the beta-band may be related to the activation of the visual system and also to the state of attention (Molteni et al., 2007). An increase in alpha- and theta-activity is usually associated with a decrease of attention and vigilance (Coelli et al., 2015), so a complex index can give a more accurate result. In other words, engagement indices, widely used in different psychophysiological studies, could enrich the field of neurocinematics, since they take into account different EEG bands, related to both activation and deactivation. Supposedly, complex or composite indices could capture differences or relationships, invisible when the EEG rhythms are studied independently (Shestyuk et al., 2019).

We also applied machine-learning methods to predict the rating of films based on EEG data and peripheral indicators, expecting that the engagement indices would be among the significant predictors of film ratings. By now, researchers predicted film scores from *Facebook* likes and number of critics (Dhir and Raj, 2018), Oscar votes (Latif and Afzal, 2016), or even facial expression (Shetty et al., 2021). Previously, machine learning was also shown to be very fruitful to recognize, via EEG signals, such mental states as, for example, engagement, workload (Berka et al., 2007; Walter et al., 2017) and emotions (Soleymani et al., 2015; Rayatdoost and Soleymani, 2018; Rayatdoost et al., 2020). This motivated us to apply machine-learning techniques to predict film rationg from EEG and peripheral signals.

In addition, previous studies have mostly used film trailers as stimuli (Boksem and Smidts, 2015; Liu et al., 2016; Christoforou et al., 2017; Wu et al., 2017, 2018; Dushantha et al., 2020). Trailers are convenient stimuli for studying as they are actively used in marketing research. However, they display a number of drawbacks, as they consist of disparate scenes of the film and rarely present a coherent narrative. In the current study, we focused on short films. According to the Academy of Motion Picture Arts and Sciences, short films are “original films that are less than 40 min long…” Short films are usually used as a test case for directors and screenwriters and can be created to raise funding for future projects because short films are much cheaper to produce. We suppose that due to their shorter duration and at the same time the integrity of the narrative, short films could be a suitable object for a psychophysiological study.

Hence, the aim of our study was to find physiological markers of viewer perception and correlate them to short film ratings. For this purpose, we recorded electroencephalography, facial electromyography (*corrugator supercilii* and *zygomaticus major*), photoplethysmography, and skin conductance that were supposed to reflect viewer engagement (Palumbo et al., 2017) and emotions (Lundqvist, 1995; Li et al., 2018; Sato et al., 2020; Madsen and Parra, 2022). As for the cardiac activity, along with heart rate, we also extracted different features of heart rate variability (HRV), which are frequently considered to reflect emotional states (Kuoppa et al., 2016; Shi et al., 2017; for a review see Zhu et al., 2019). Finally, we used machine learning to predict both the exact rating of each film and classify them as low or high rated.

## Materials and methods

### Sample

Twenty-one healthy volunteers (76.19% females) participated in the experiment in exchange for a monetary reward (an equivalent of 20 USD at purchasing parity power in 2021). Their mean age was 22.5, SD = 4.0. The study was carried out in accordance with the Declaration of Helsinki and was approved by the local research ethics committee (#52, 14.01.2019). Each participant provided written consent for his or her participation in the study.

### Stimuli

We selected eight short films with different levels of scores on the *Kinopoisk*, a film rating database. The mean score was 7.5 (SD = 0.5, min = 6.8, max = 8.1) on a scale of 1 to 10. The number of ratings for each video was over 800, with an average of 5,400 ratings. We removed the titles, so the subjects could watch only the films. The mean duration was 6 min 4 s; the range was from 4 min 21 s to 7 min 25 s (Table 1).

**Table 1:** The description of short films used in the study.

Title	Year	Duration, s	Genre	Director	Rating page (consulted 1/3/2021)
*One-Minute Time Machine*	2014	318	Comedy	D. Avery	https://www.kinopoisk.ru/film/864243/
*Star*	2001	395	Comedy	G. Ritchie	https://www.kinopoisk.ru/film/12201/
*The Flying Car*	2002	364	Comedy	K. Smith	https://www.kinopoisk.ru/film/328034/
*The Expert*	2014	445	Comedy	L. Beinerts	https://www.kinopoisk.ru/film/838922/
*One Hundredth of a Second*	2006	261	Drama	S. Jacobson	https://www.kinopoisk.ru/film/272683/
*The Gift*	2010	391	Drama	I. Petukhov	https://www.kinopoisk.ru/film/841213/
*Aningaaq*	2013	346	Drama	J. Cuarón	https://www.kinopoisk.ru/film/788239/
*Cargo*	2013	394	Drama	B. Howling, Y. Ramke	https://www.kinopoisk.ru/film/756665/

### Procedure

Participants were informed that they would have to watch and evaluate a number of short films. Each participant watched short films on a 31.5-inch computer screen in a random order. After watching each film, participants were asked “to rate the film” on a scale of 1 to 10 (where 10 meant the best grade, following *Kinopoisk* or *IMDb* scales). There was a rest period for 60 s between the evaluation offset and a new film onset. Participants were asked not to move and blink much, because the rest periods were used in the analyses as well (see below). The procedure was programmed in *PsychoPy* (Peirce, 2008).

### Data collection

To record and amplify physiological signals, we used ActiChamp equipment (Brain Products, Germany). The signal recording frequency was 1,000 Hz. EEG signals were recorded from 18 active electrodes (F7, F3, Fz, F4, F8, T7, C3, Cz, C4, T8, P8, P4, Pz, P3, P7, O1, Oz, O2), according to the 10–20 system (Jasper, 1958). The impedance of each electrode was kept below 15 kΩ. Tp9 and Tp10 electrodes were used as an online reference. Vertical eye movements were recorded with one electrode, which was placed on the orbicularis oculi muscle under the right eye. A photoplethysmograph was put on the middle finger of the left hand. Electromyographic activity of *zygomaticus major* (the “smiling” muscle) and *corrugator supercilii* (the “frowning” muscle) was recorded by placing 4-mm Ag/AgCl surface electrodes (Fridlund and Cacioppo, 1986). Although it was shown that the left side of the face is more sensitive to corresponding emotions (Dimberg and Petterson, 2000), due to technical problems we recorded EMG from the right side. Skin conductance was measured by placing two Ag/AgCl surface electrodes on the index and ring fingers of the left hand (non-dominant for all participants).

### Data reduction

The EEG preprocessing was conducted in MNE Python (Gramfort, 2013). The raw EEG was downsampled from 1,000 Hz to 125 Hz to reduce computational complexity and filtered with lower-pass edge of 0.05 and upper-pass of 30 Hz. We decided to exclude the gamma-activity, since recent studies show that it is not possible to completely clear the signal from muscle activity in the gamma-band. And also that data contamination from muscle activity in the gamma band >30 Hz over the entire scalp is higher than in the beta band. Moreover, the analysis of independent components provides effective clipping of EMG in EEG beta activity in almost all leads, but not in gamma (Pope et al., 2022). After that, we interpolated bad channels by fitting PyPrep Pipeline with RANSAC method (Bigdely-Shamlo et al., 2015). To correct EEG for eye blinks, we ran ICA decomposition from MNE.ICA module. To reject muscle artefacts, we deleted intervals (about 2% of the data) where the *z*-score was greater than 10. We estimated power spectral density using Welch’s method in Yasa SciPy welch (Vallat and Walker, 2021). We computed the median power of the EEG in theta (4–8 Hz), alpha (8–12 Hz), and beta (12–30 Hz) bands in the one-second window (125 samples) with a 50% overlap. We also got ratios of theta, alpha, and beta bands within each trial (one film, one subject). In addition, we calculated the engagement index as beta/(alpha+theta) and beta/alpha (Pope et al., 1995; Freeman et al., 1999), arousal index, (beta_F3_ + beta_F4_)/(alpha_F3_ + alpha_F4_), and valence index, (alpha_F4_/beta_F4_) - (alpha_F3_/beta_F3_), where positive values mean positive emotions (Giraldo and Ramirez, 2013). For each variable, we subtracted the baseline (the mean value of 60-s rest period before each film) from the value of each trial. For machine-learning purposes, following an increasing trend towards the use of complexity analysis in quantifying neural activity, we additionally calculated brain entropy and complexity measures (Lau et al., 2022). Using neurokit2 (Makowski et al., 2021), we extracted Petrosian fractal dimension (PFD), differential entropy (DE), Katz’s fractal dimension (KFD), Sevcik fractal dimension (SFD), permutation entropy (PEn), Shannon entropy (ShanEn), spectral entropy, singular value decomposition entropy (SVDEn), Fisher information (FI), Hjorth’s complexity (Hjorth), relative roughness (RR) for each EEG channel and each film (Rahman et al., 2021).

Photoplethysmograms were processed with HeartPy, Python heart rate analysis toolkit (Van Gent et al., 2019). They were filtered using a Hampel filter with filter size parameter set on 6, that means that three data points on each side were taken to detect outliers and correct the signal. For each film, we extracted variables thought to reflect emotional states (Kuoppa et al., 2016; Shi et al., 2017; Zhu et al., 2019): mean heart rate (HR) and different characteristics of heart rate variability (HRV): the standard deviation of NN intervals (SDNN), the root mean square of the successive differences (RMSSD), the standard deviation between successive differences (SDSD), the proportion of NN20 and NN50 (pNN20 and pNN50), the median absolute deviation of RR intervals (MAD), and SD1, SD2, SD1/SD2 of Poincaré plot. For each variable, we subtracted the baseline (the mean value of 60-s rest period before each film) from the value of each trial.

Zygomaticus and corrugator EMG activity was processed in MNE-Python. We applied the FIR filter with a lower–pass of 10 Hz and upper-pass of 350 Hz, took absolute values of the signal and averaged it within each trial. Then we subtracted the baseline (the mean value of 60-s rest period before each film) from the EMG value of each trial.

Skin conductance was processed with Neurokit2 (Makowski et al., 2021). We excluded the tonic component, detected skin conductance responses, and extracted amplitudes of all peaks. For each trial, we summed all the peak amplitudes and divided by time in order to correct for different epoch length.

### Data analysis

We compared film ratings and somatic and vegetative variables between genres (comedies/dramas), using *t*-tests for paired samples with Cohen’s *d* as an effect size measure. Then we correlated film ratings with all physiological variables. In the analysis of EEG, Benjamini-Hochberg correction was applied for multiple correlations for each individual channel.

To predict film ratings, based on physiological data, we used all above-mentioned features. After this, we removed outliers defined as >3 SD or < −3 SD by column in our matrix. After this, we imputed missed values with a multivariate imputation by chained equations in which the specification occurs on at the variable level, excluding artificial correlations between them (mice; Van Buuren and Groothuis-Oudshoorn, 2011). The final matrix consisted of 168 cases (8 films × 21 subjects) and 522 columns (519 physiological features (see Supplementary materials), film, subject and rating). For each prediction, we extracted 15 most important physiological features, which then were used for training and prediction. The importance of a feature was computed as the reduction of the criterion brought by that feature. It is also known as the Gini importance (Nembrini et al., 2018). Thus, the final matrices were always 168 × 18 (15 physiological features, film, subject and rating). To predict continuous ratings (from 1 to 10), we applied CatBoost (CatBoost Python package; Prokhorenkova et al., 2018) and support vector regression (with Scikit-learn Python package). Additionally, we ranked all the ratings within each subject as low or high rated to apply binary classification with logistic regression, KNN, decision tree, CatBoost, support vector classification (with Scikit-learn Python package). To compute metrics on regression and classification, we used *k*-fold cross-validation. We applied the leave-one-film-out strategy; hence, for each of the eight films, the data collected from all participants who watched seven of them were utilized for training. Meanwhile, the other film was designated for testing purposes, and to forecast the ratings of all subjects for the eighth film. This process was carried out independently for each film, resulting in eight separate predictions (Kramer, 2016). In other words, we expected to predict the film ratings (from 1 to 10) or ranks (low/high) of a film, based on the EEG and peripheral signals data of this film and EEG and peripheral signals and ratings or ranks of seven other films. This might have an applied significance in future attempts to predict ratings at the film production stage. In simple words, a studio could collect physiological databases during film perception and after having shown some films and asked for ratings to a sample of subjects in a neuroscientific laboratory, they could predict subjects’ ratings of new films.

## Results

First, we analysed whether genres provoked different reactions. Film ratings did not differ depending on genres, *t* (20) = 0.67, *p* = 0.50, *M* ± SD_dramas_ = 6.12 ± 2.43, *M* ± SD_comedies_ = 6.37 ± 2.40. Corrugator supercilii activity was larger while watching dramas, *t* (20) = 3.25, *p* = 0.004, *d* = 0.71. Zygomaticus major activity was larger while watching comedies, *t* (20) = 2.12, *p* = 0.047, *d* = 0.46. All other somatic and vegetative variables did not show differences between comedies and dramas, *t*s < 1.6, *p*s > 0.08.

**Figure 1:**
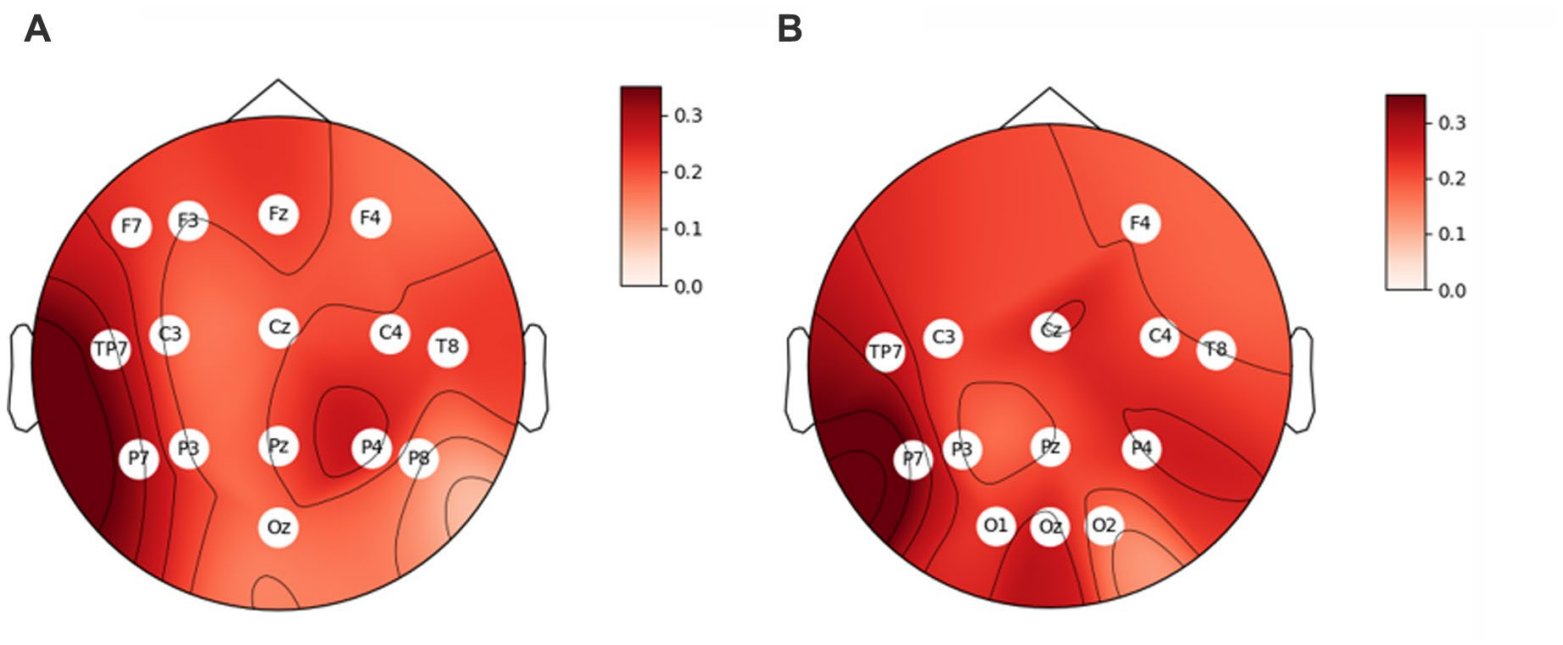
Correlation coefficients between film ratings and the engagement index, **(A)** beta/(alpha+theta), and **(B)** beta/alpha. The sensors where the correlation is significant (*p*s < 0.05) are indicated with their titles.

Second, we subjected all physiological variables to correlation analysis with the film ratings. Of all somatic and vegetative markers, only three were related to film ratings. *Zygomaticus major* activity positively correlated to film ratings, *r* = 0.26, *p* = 0.001. Also, PNN50 and SD1/SD2 (indices of HRV) as well positively correlated to film ratings, *r* = 0.18, *p* = 0.019 and *r* = 0.16, *p* = 0.043. Correlation analysis between EEG rhythms and film ratings yielded some significant results. Thus, the engagement index, beta/(alpha+theta), correlated positively with film ratings in the majority of sensors (Figure 1A). The higher the index was, the larger self-reported value was. We received a similar pattern when we calculated the engagement index as beta/alpha. It also correlated positively with film ratings in the majority of sensors (Figure 1B). Valence and arousal indices also positively correlated to film ratings (*r* = 0.21, *p* = 0.010 and *r* = 0.24, *p* = 0.003, respectively). Of note, the engagement index beta/(alpha+theta) positively correlated to the arousal index in all sensors (0.31 < *r*s < 0.80, all *p*s < 0.001), besides O1, Oz and O2, but to the valence index only in Cz, negatively (*r* = − 0.28, *p* = 0.007). The engagement index beta/alpha positively correlated to the arousal index in all sensors (0.29 < *r*s < 0.88, all *p*s < 0.001), and negatively to the valence index only in Cz (*r* = − 0.23, *p* = 0.004).

We also separately correlated the power of each band to film ratings and did not find any correlation (all *p*s > 0.05). Finally, within each subject we split films into low and high rated halves. Mean ratings were 4.55 and 8.02, *t* = 13.34, *p* < 0.001; but the interaction genre × half (low/high) was not significant, *p* = 0.57. However, we found no significant difference in any physiological measure between halves (*t*s < 1.79, *p*s > 0.08).

**Figure 2:**
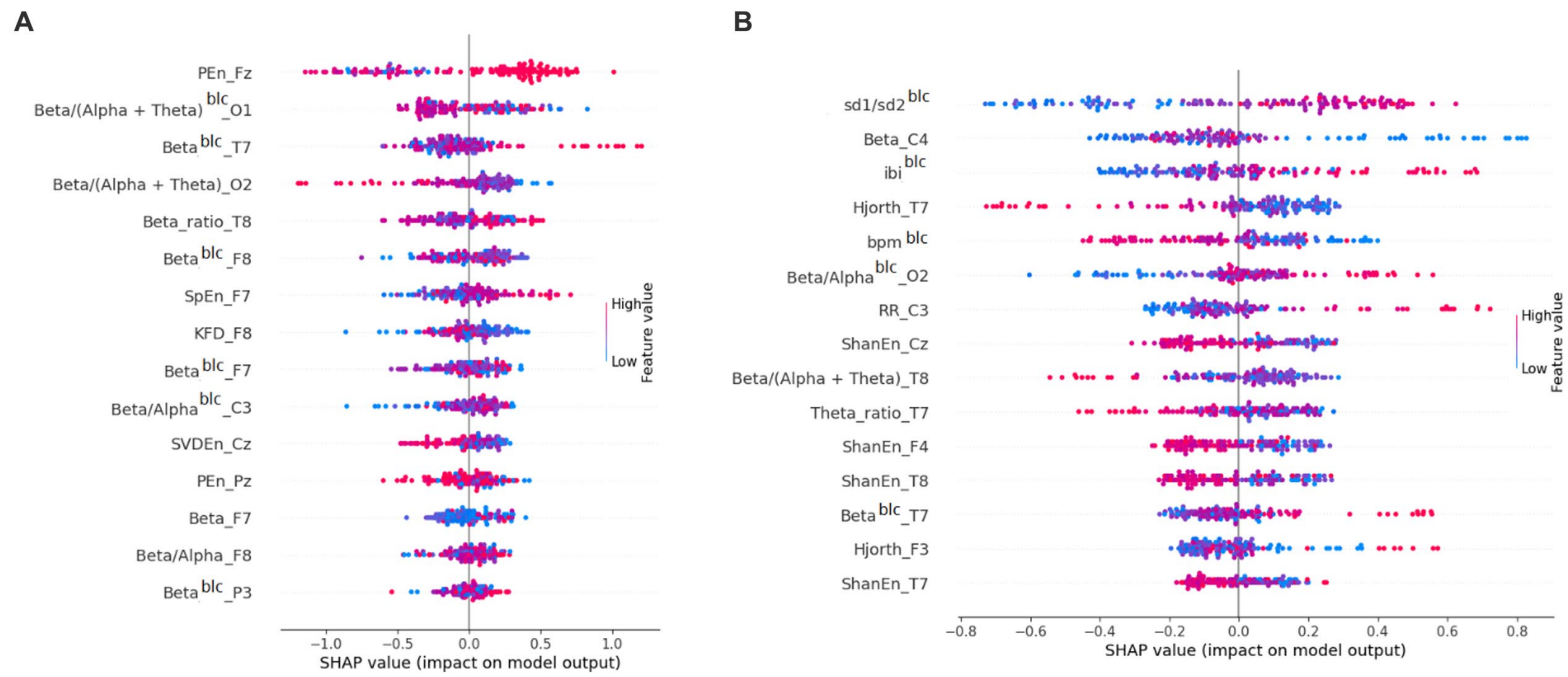
Importance of features for prediction in **(A)** CatBoost regression for Film 1 and **(B)** CatBoost classification for Film 6. Shapley Additive Explanation (SHAP) values attribute to each feature the change in the expected model prediction when conditioning on that feature (Lundberg and Lee, 2017). blc, baseline-corrected.

Third, we attempted to predict (using cross-validation) film ratings based on physiological data. We predicted the rating of each film based on the physiological data of all subjects recorded while watching the other films. As an example, the 15 variables with their importance in CatBoost regression for Film 1 can be found in Figure 2. For both models applied, the mean absolute prediction error (MAPE) was 0.53. The statistics of predictions for each film can be found in Table 2.

**Table 2:** Statistics of rating predictions for each of 8 films, based on seven other films.

Film	Observed rating	CatBoost			SVR		
		MAPE	MAE	Predicted	MAPE	MAE	Predicted
1	7.52	0.26	1.96	6.13	0.30	2.04	6.45
2	6.50	0.37	1.85	6.30	0.34	1.82	6.29
3	4.86	0.62	2.05	6.41	0.72	2.25	6.76
4	6.62	0.49	2.52	6.10	0.61	2.44	6.60
5	6.52	0.86	2.39	6.21	0.73	2.06	6.28
6	6.10	0.39	1.84	6.15	0.33	1.37	6.25
7	6.52	0.38	2.17	6.19	0.36	1.98	6.01
8	5.33	0.84	2.36	6.64	0.85	2.17	6.70
Mean of 8 films		0.53	2.14		0.53	2.02	

We then tried the binary classification of ratings. That is, within each subject we split films into low and high rated halves. After this, for each film we predicted whether it would receive a low or high rating by each subject, based on the binary ratings of all other films (Table 3). As an example, the 15 variables with their importance in CatBoost classification for Film 6 can be found in Figure 2. Logistic regression turned out to be the best predictive model. The mean area under the curve was 0.62 (with 0.50 being the random value). The best area under the curve (0.71) was found for Film 6. Other models yielded lower values of the area under the curve (0.51–0.59).

**Table 3:** Statistics of binary predictions for each of 8 films, based on seven other films, and the mean values for different models.

	Logistic regression			CatBoost			KNN			Decision trees			SVC		
Film	Acc.	F1	AUC	Acc.	F1	AUC	Acc.	F1	AUC	Acc.	F1	AUC	Acc.	F1	AUC
1	0.52	0.55	0.62	0.52	0.58	0.57	0.57	0.67	0.55	0.38	0.48	0.37	0.29	0.35	0.30
2	0.60	0.60	0.61	0.70	0.67	0.70	0.40	0.33	0.39	0.65	0.59	0.64	0.60	0.56	0.60
3	0.62	0.43	0.67	0.52	0.38	0.61	0.57	0.31	0.54	0.52	0.38	0.61	0.62	0.33	0.57
4	0.52	0.55	0.53	0.52	0.62	0.50	0.52	0.50	0.54	0.52	0.50	0.54	0.48	0.56	0.46
5	0.71	0.80	0.67	0.57	0.73	0.50	0.52	0.69	0.46	0.57	0.64	0.56	0.52	0.50	0.54
6	0.70	0.67	0.71	0.70	0.57	0.67	0.55	0.53	0.56	0.55	0.53	0.56	0.55	0.61	0.60
7	0.48	0.52	0.48	0.57	0.61	0.58	0.38	0.38	0.40	0.33	0.36	0.34	0.43	0.40	0.47
8	0.62	0.64	0.67	0.62	0.56	0.62	0.62	0.56	0.62	0.52	0.58	0.59	0.62	0.50	0.60
Mean	0.60	0.60	0.62	0.59	0.59	0.59	0.52	0.50	0.51	0.51	0.51	0.53	0.51	0.47	0.51

acc. – accuracy, AUC – area under the curve.

## Discussion

The aim of our study was to explore physiological markers of short film perception and correlate them to film ratings. For this purpose, we measured electroencephalography, facial electromyography, photoplethysmography and skin conductance in 21 participants, while watching 8 short films (4 dramas and 4 comedies). We also used machine learning to predict the exact rating of each film and to classify each film as low or high rated.

First, we simply compared ratings and all physiological variables between two genres. *Corrugator supercilii* (the “frowning” muscle) activity was larger in response to dramas, while comedies provoked an increase in *zygomaticus major* (the “smiling” muscle) EMG. These findings are in accordance with the previous studies, which showed that unpleasant stimuli evoke an increase in *corrugator supercilii* activity, whereas *zygomaticus major* is activated by pleasant stimuli (Dimberg and Karlsson, 1997; Bullack et al., 2018). At the same time, heart rate and skin conductance did not differ. This may mean that both comedies and dramas evoked the same level of arousal, but with the opposite valence (Bradley et al., 2001). In other words, comedies were perceived positively, while dramas evoked negative emotions, but the same level of physiological arousal.

Curiously, ratings between genres did not differ, which may mean that they might reflect arousal rather than valence. We then split films into low and high rated halves (within each subject), but found no significant difference in any physiological measure between the halves. Also, the interaction between halves and genres did not have a significant effect on the ratings, which would mean that comedies and dramas were distributed equally between halves. In other words, both comedies and dramas were in low and high rated halves. This may explain that many films and genres are not perceived as pleasant (like horror), but attract the attention of viewers, presumably, due to the level of arousal. This is consistent with the suggestion that for many viewers, arousal itself may be an important reason for watching, like in the case of horrors or tragedies (Martin, 2019). Thus, in a study by Vecchiato et al. (2009), skin conductance (a marker of arousal) was not sensitive to differentiate TV commercials, seemingly because of the equally high level of arousal.

Second, correlation analysis between EEG rhythms and film ratings showed that the engagement index (calculated as beta/(alpha+theta) or beta/alpha), correlated positively with the ratings in the majority of sensors. The higher the index was, the larger self-reported value (film rating) was. We admit that the correlations were weak, however consistent throughout the scalp. At the same time, EEG indices of valence and arousal also correlated to film ratings. In other words, positive and arousing films (based on EEG) were evaluated with a higher rating. Also, engagement indices correlated to the arousal index. These findings confirm previous studies that revealed that engagement index reflects arousal rather than valence. Thus, Chaouachi et al. (2010) found that engagement index correlated with arousal, but not with valence in an educational process. McMahan et al. (2015) showed that the engagement index differentiated low and high intensity video games. It was positively related to arousal, and, at the same time, negatively to valence. In other words, the engagement index increased in response to a more arousing and unpleasant video game event. Maran et al. (2017) also concluded that engaging and effective educational material should induce arousal states of different valence, both aversive and appetitive.

However, besides arousal, the EEG index of valence and *zygomaticus major* activity positively correlated to film ratings, as well as PNN50 and SD1/SD2 (HRV variables). This is in accordance with the previous findings on the frontal EEG asymmetry (Davidson, 2004), indicating the role of the left hemisphere in positive emotions. As for HRV, although being a controversial marker of affective states, it was found to reflect valence as well. Thus, Shi et al. (2017) revealed its increase during happy states in comparison to sad ones. On the contrary, Kuoppa et al. (2016) found a lower HRV in response to positive food, compared to negative food, but no difference for non-edible stimuli. Nevertheless, our results coincide with the data of Vecchiato et al. (2011) who showed that spectral EEG frontal asymmetries correlate with the experienced pleasantness of TV commercial advertisements. In this vein, Shestyuk et al. (2019), found a correlation between frontal asymmetry (pleasantness) and TV viewership (number of viewers). In addition, our data partly replicated the findings of Sato et al. (2020) who showed correlations between *corrugator* and *zygomaticus* EMG and the valence of films. Thus, at the same time, film ratings reflect valence and arousal experienced during perception. It is worth noting, that we deliberately avoided the typical affective self-report scales of valence and arousal. We wanted to replicate the scales used in the film industry of mere “rating,” which, as we understand, does not equal valence, because even for films in the same genres and equal user ratings, the emotions of the audience can be radically different (Topal and Ozsoyoglu, 2016). We had a concern that the usage of all three scales (film rating, valence, arousal) could impact the perception of the “film rating” scale, that is, this could have suggested subjects to consider the nature of “film rating.”

Third, we tested several machine learning models in order to predict ratings, based on the physiological data. We predicted the exact rating of each film and classified each film as low or high rated. As for prediction of exact ratings, the MAPE was 0.55 both for CatBoost and SVR. When we classified films as low or high rated, the best area under the curve equalled 0.62 in the case of logistic regression. Interestingly, in a study by Dhir and Raj (2018), where they predicted film ratings, based on *Facebook* likes and number of critics, the prediction quality was low (F1 = 0.59), although their sample consisted of 5,043 films. Much better results on 2000 films were obtained by Latif and Afzal (2016), who used budget, genre, critics, Oscar votes to predict film ratings (ROC area = 0.93). This discrepancy can lie in the difference of films used in the study. Due to the restrictions of psychophysiological laboratory and our experimental plan, we could present only eight films, in comparison to the studies relied only on open data from large samples.

A future study could involve much more films and evaluated models with and without physiological data. To resolve this and present more films, future studies could be organised so that different subjects could watch some overlapping subsamples of films. Another possibility to expand this line of research would be to ask subjects to indicate a “dynamic valence/arousal rating” during the whole viewing (Nummenmaa et al., 2012). These time-series could then be correlated to physiological markers within each trial or on average. This would allow finding crucial scenes in films. We had a concern that the usage of different self-report scales (film rating, valence, arousal) could have impacted viewer perception. Therefore, future large-scale studies could go deeper into the question of the relationship between these scales in order to understand what psychological phenomena lie behind “film rating.” Our physiological exploration implies that it can be a fusion of high arousal and different valence, positive valence being more important on average. Nevertheless, this would depend on genre, since some films inducing negative emotions, like horror (Zillmann, 1996) or sadness (Oliver, 1993), also obtain very high ratings.

Another limitation of our study lies in the recording of EMG from the right side the face. Previously, it was shown that the left side of the face is more sensitive to basic emotions. Thus, Dimberg and Petterson (2000) showed that *corrugator supercilii* and *zygomaticus major* were more activated on the left side of face, while expressing anger and happiness, respectively. This pattern was then confirmed by Zhou and Hu (2004). This difference could reflect the right hemisphere dominance in emotional expression. Supposedly, future studies in the field of neurocinematics could apply EMG sensors to both hemifaces for a more detailed analysis.

To conclude, we revealed that the engagement, valence and arousal indices of EEG, as well as the *zygomaticus* activity and some HRV variables, positively correlated to short film ratings given by our subjects. Central and peripheral markers, thus, reflect viewer ratings and can predict them to a certain extent, as we showed using machine learning.

## Data availability statement

The raw data supporting the conclusions of this article will be made available by the authors, without undue reservation. All the code for processing and machine learning has been uploaded to https://github.com/avenator/EEG-FILMS.

## Ethics statement

The studies involving human participants were reviewed and approved by Institutional Review Board of HSE University (No. 52, 14.01.2019). The patients/participants provided their written informed consent to participate in this study.

## Author contributions

VK: design, analysis, and writing. DS: recording and writing. NR: analysis. All authors contributed to the article and approved the submitted version.

## Funding

This work was supported by the International Laboratory of Social Neurobiology ICN HSE RF Government Grant Ag. No. 075–15–2022-1037 and was carried out using Unique Scientific Installation of the National Research University Higher School of Economics “Automated System for Non-Invasive Brain Stimulation with the Possibility of Synchronous Registration of Brain Biocurrents and Eye Movement Tracking”.

## Conflict of interest

The authors declare that the research was conducted in the absence of any commercial or financial relationships that could be construed as a potential conflict of interest.

## Publisher’s note

All claims expressed in this article are solely those of the authors and do not necessarily represent those of their affiliated organizations, or those of the publisher, the editors and the reviewers. Any product that may be evaluated in this article, or claim that may be made by its manufacturer, is not guaranteed or endorsed by the publisher.
