# Supplementary material for: EEG and peripheral markers of viewer ratings: a study of short films
Source: Front Neurosci. 2023 Jun 12;17:1148205. doi: 10.3389/fnins.2023.1148205 (PMC10291053; doi:10.3389/fnins.2023.1148205)
Supplement: Supplementary file 1 [file Table_1.DOCX]

S-Table 1. All extracted physiological features, used in machine learning.

| Alpha_Fz |
| --- |
| Alpha_F3 |
| Alpha_F7 |
| Alpha_C3 |
| Alpha_T7 |
| Alpha_Pz |
| Alpha_P3 |
| Alpha_P7 |
| Alpha_O1 |
| Alpha_Oz |
| Alpha_O2 |
| Alpha_P4 |
| Alpha_P8 |
| Alpha_Cz |
| Alpha_C4 |
| Alpha_T8 |
| Alpha_F4 |
| Alpha_F8 |
| Alpha_ratio_Fz |
| Alpha_ratio_F3 |
| Alpha_ratio_F7 |
| Alpha_ratio_C3 |
| Alpha_ratio_T7 |
| Alpha_ratio_Pz |
| Alpha_ratio_P3 |
| Alpha_ratio_P7 |
| Alpha_ratio_O1 |
| Alpha_ratio_Oz |
| Alpha_ratio_O2 |
| Alpha_ratio_P4 |
| Alpha_ratio_P8 |
| Alpha_ratio_Cz |
| Alpha_ratio_C4 |
| Alpha_ratio_T8 |
| Alpha_ratio_F4 |
| Alpha_ratio_F8 |
| Alpha_watch-relax_Fz |
| Alpha_watch-relax_F3 |
| Alpha_watch-relax_F7 |
| Alpha_watch-relax_C3 |
| Alpha_watch-relax_T7 |
| Alpha_watch-relax_Pz |
| Alpha_watch-relax_P3 |
| Alpha_watch-relax_P7 |
| Alpha_watch-relax_O1 |
| Alpha_watch-relax_Oz |
| Alpha_watch-relax_O2 |
| Alpha_watch-relax_P4 |
| Alpha_watch-relax_P8 |
| Alpha_watch-relax_Cz |
| Alpha_watch-relax_C4 |
| Alpha_watch-relax_T8 |
| Alpha_watch-relax_F4 |
| Alpha_watch-relax_F8 |
| Beta_Fz |
| Beta_F3 |
| Beta_F7 |
| Beta_C3 |
| Beta_T7 |
| Beta_Pz |
| Beta_P3 |
| Beta_P7 |
| Beta_O1 |
| Beta_Oz |
| Beta_O2 |
| Beta_P4 |
| Beta_P8 |
| Beta_Cz |
| Beta_C4 |
| Beta_T8 |
| Beta_F4 |
| Beta_F8 |
| Beta/(Alpha+Theta)_Fz |
| Beta/(Alpha+Theta)_F3 |
| Beta/(Alpha+Theta)_F7 |
| Beta/(Alpha+Theta)_C3 |
| Beta/(Alpha+Theta)_T7 |
| Beta/(Alpha+Theta)_Pz |
| Beta/(Alpha+Theta)_P3 |
| Beta/(Alpha+Theta)_P7 |
| Beta/(Alpha+Theta)_O1 |
| Beta/(Alpha+Theta)_Oz |
| Beta/(Alpha+Theta)_O2 |
| Beta/(Alpha+Theta)_P4 |
| Beta/(Alpha+Theta)_P8 |
| Beta/(Alpha+Theta)_Cz |
| Beta/(Alpha+Theta)_C4 |
| Beta/(Alpha+Theta)_T8 |
| Beta/(Alpha+Theta)_F4 |
| Beta/(Alpha+Theta)_F8 |
| Beta/Alpha_Fz |
| Beta/Alpha_F3 |
| Beta/Alpha_F7 |
| Beta/Alpha_C3 |
| Beta/Alpha_T7 |
| Beta/Alpha_Pz |
| Beta/Alpha_P3 |
| Beta/Alpha_P7 |
| Beta/Alpha_O1 |
| Beta/Alpha_Oz |
| Beta/Alpha_O2 |
| Beta/Alpha_P4 |
| Beta/Alpha_P8 |
| Beta/Alpha_Cz |
| Beta/Alpha_C4 |
| Beta/Alpha_T8 |
| Beta/Alpha_F4 |
| Beta/Alpha_F8 |
| Beta_ratio_Fz |
| Beta_ratio_F3 |
| Beta_ratio_F7 |
| Beta_ratio_C3 |
| Beta_ratio_T7 |
| Beta_ratio_Pz |
| Beta_ratio_P3 |
| Beta_ratio_P7 |
| Beta_ratio_O1 |
| Beta_ratio_Oz |
| Beta_ratio_O2 |
| Beta_ratio_P4 |
| Beta_ratio_P8 |
| Beta_ratio_Cz |
| Beta_ratio_C4 |
| Beta_ratio_T8 |
| Beta_ratio_F4 |
| Beta_ratio_F8 |
| Beta_watch-relax_Fz |
| Beta_watch-relax_F3 |
| Beta_watch-relax_F7 |
| Beta_watch-relax_C3 |
| Beta_watch-relax_T7 |
| Beta_watch-relax_Pz |
| Beta_watch-relax_P3 |
| Beta_watch-relax_P7 |
| Beta_watch-relax_O1 |
| Beta_watch-relax_Oz |
| Beta_watch-relax_O2 |
| Beta_watch-relax_P4 |
| Beta_watch-relax_P8 |
| Beta_watch-relax_Cz |
| Beta_watch-relax_C4 |
| Beta_watch-relax_T8 |
| Beta_watch-relax_F4 |
| Beta_watch-relax_F8 |
| Beta_watch/(Alpha_watch+Theta_relax)-Beta_relax/(Alpha_relax+Theta_relax)_Fz |
| Beta_watch/(Alpha_watch+Theta_relax)-Beta_relax/(Alpha_relax+Theta_relax)_F3 |
| Beta_watch/(Alpha_watch+Theta_relax)-Beta_relax/(Alpha_relax+Theta_relax)_F7 |
| Beta_watch/(Alpha_watch+Theta_relax)-Beta_relax/(Alpha_relax+Theta_relax)_C3 |
| Beta_watch/(Alpha_watch+Theta_relax)-Beta_relax/(Alpha_relax+Theta_relax)_T7 |
| Beta_watch/(Alpha_watch+Theta_relax)-Beta_relax/(Alpha_relax+Theta_relax)_Pz |
| Beta_watch/(Alpha_watch+Theta_relax)-Beta_relax/(Alpha_relax+Theta_relax)_P3 |
| Beta_watch/(Alpha_watch+Theta_relax)-Beta_relax/(Alpha_relax+Theta_relax)_P7 |
| Beta_watch/(Alpha_watch+Theta_relax)-Beta_relax/(Alpha_relax+Theta_relax)_O1 |
| Beta_watch/(Alpha_watch+Theta_relax)-Beta_relax/(Alpha_relax+Theta_relax)_Oz |
| Beta_watch/(Alpha_watch+Theta_relax)-Beta_relax/(Alpha_relax+Theta_relax)_O2 |
| Beta_watch/(Alpha_watch+Theta_relax)-Beta_relax/(Alpha_relax+Theta_relax)_P4 |
| Beta_watch/(Alpha_watch+Theta_relax)-Beta_relax/(Alpha_relax+Theta_relax)_P8 |
| Beta_watch/(Alpha_watch+Theta_relax)-Beta_relax/(Alpha_relax+Theta_relax)_Cz |
| Beta_watch/(Alpha_watch+Theta_relax)-Beta_relax/(Alpha_relax+Theta_relax)_C4 |
| Beta_watch/(Alpha_watch+Theta_relax)-Beta_relax/(Alpha_relax+Theta_relax)_T8 |
| Beta_watch/(Alpha_watch+Theta_relax)-Beta_relax/(Alpha_relax+Theta_relax)_F4 |
| Beta_watch/(Alpha_watch+Theta_relax)-Beta_relax/(Alpha_relax+Theta_relax)_F8 |
| Beta_watch/(Alpha_watch+Theta_watch)_Fz |
| Beta_watch/(Alpha_watch+Theta_watch)_F3 |
| Beta_watch/(Alpha_watch+Theta_watch)_F7 |
| Beta_watch/(Alpha_watch+Theta_watch)_C3 |
| Beta_watch/(Alpha_watch+Theta_watch)_T7 |
| Beta_watch/(Alpha_watch+Theta_watch)_Pz |
| Beta_watch/(Alpha_watch+Theta_watch)_P3 |
| Beta_watch/(Alpha_watch+Theta_watch)_P7 |
| Beta_watch/(Alpha_watch+Theta_watch)_O1 |
| Beta_watch/(Alpha_watch+Theta_watch)_Oz |
| Beta_watch/(Alpha_watch+Theta_watch)_O2 |
| Beta_watch/(Alpha_watch+Theta_watch)_P4 |
| Beta_watch/(Alpha_watch+Theta_watch)_P8 |
| Beta_watch/(Alpha_watch+Theta_watch)_Cz |
| Beta_watch/(Alpha_watch+Theta_watch)_C4 |
| Beta_watch/(Alpha_watch+Theta_watch)_T8 |
| Beta_watch/(Alpha_watch+Theta_watch)_F4 |
| Beta_watch/(Alpha_watch+Theta_watch)_F8 |
| Beta_watch/Alpha_watch_Fz |
| Beta_watch/Alpha_watch_F3 |
| Beta_watch/Alpha_watch_F7 |
| Beta_watch/Alpha_watch_C3 |
| Beta_watch/Alpha_watch_T7 |
| Beta_watch/Alpha_watch_Pz |
| Beta_watch/Alpha_watch_P3 |
| Beta_watch/Alpha_watch_P7 |
| Beta_watch/Alpha_watch_O1 |
| Beta_watch/Alpha_watch_Oz |
| Beta_watch/Alpha_watch_O2 |
| Beta_watch/Alpha_watch_P4 |
| Beta_watch/Alpha_watch_P8 |
| Beta_watch/Alpha_watch_Cz |
| Beta_watch/Alpha_watch_C4 |
| Beta_watch/Alpha_watch_T8 |
| Beta_watch/Alpha_watch_F4 |
| Beta_watch/Alpha_watch_F8 |
| Beta_watch/Alpha_watch-Beta_relax/Alpha_relax_Fz |
| Beta_watch/Alpha_watch-Beta_relax/Alpha_relax_F3 |
| Beta_watch/Alpha_watch-Beta_relax/Alpha_relax_F7 |
| Beta_watch/Alpha_watch-Beta_relax/Alpha_relax_C3 |
| Beta_watch/Alpha_watch-Beta_relax/Alpha_relax_T7 |
| Beta_watch/Alpha_watch-Beta_relax/Alpha_relax_Pz |
| Beta_watch/Alpha_watch-Beta_relax/Alpha_relax_P3 |
| Beta_watch/Alpha_watch-Beta_relax/Alpha_relax_P7 |
| Beta_watch/Alpha_watch-Beta_relax/Alpha_relax_O1 |
| Beta_watch/Alpha_watch-Beta_relax/Alpha_relax_Oz |
| Beta_watch/Alpha_watch-Beta_relax/Alpha_relax_O2 |
| Beta_watch/Alpha_watch-Beta_relax/Alpha_relax_P4 |
| Beta_watch/Alpha_watch-Beta_relax/Alpha_relax_P8 |
| Beta_watch/Alpha_watch-Beta_relax/Alpha_relax_Cz |
| Beta_watch/Alpha_watch-Beta_relax/Alpha_relax_C4 |
| Beta_watch/Alpha_watch-Beta_relax/Alpha_relax_T8 |
| Beta_watch/Alpha_watch-Beta_relax/Alpha_relax_F4 |
| Beta_watch/Alpha_watch-Beta_relax/Alpha_relax_F8 |
| DiffEn_Fz |
| DiffEn_F3 |
| DiffEn_F7 |
| DiffEn_C3 |
| DiffEn_T7 |
| DiffEn_Pz |
| DiffEn_P3 |
| DiffEn_P7 |
| DiffEn_O1 |
| DiffEn_Oz |
| DiffEn_O2 |
| DiffEn_P4 |
| DiffEn_P8 |
| DiffEn_Cz |
| DiffEn_C4 |
| DiffEn_T8 |
| DiffEn_F4 |
| DiffEn_F8 |
| FI_Fz |
| FI_F3 |
| FI_F7 |
| FI_C3 |
| FI_T7 |
| FI_Pz |
| FI_P3 |
| FI_P7 |
| FI_O1 |
| FI_Oz |
| FI_O2 |
| FI_P4 |
| FI_P8 |
| FI_Cz |
| FI_C4 |
| FI_T8 |
| FI_F4 |
| FI_F8 |
| Hjorth_Fz |
| Hjorth_F3 |
| Hjorth_F7 |
| Hjorth_C3 |
| Hjorth_T7 |
| Hjorth_Pz |
| Hjorth_P3 |
| Hjorth_P7 |
| Hjorth_O1 |
| Hjorth_Oz |
| Hjorth_O2 |
| Hjorth_P4 |
| Hjorth_P8 |
| Hjorth_Cz |
| Hjorth_C4 |
| Hjorth_T8 |
| Hjorth_F4 |
| Hjorth_F8 |
| KFD_Fz |
| KFD_F3 |
| KFD_F7 |
| KFD_C3 |
| KFD_T7 |
| KFD_Pz |
| KFD_P3 |
| KFD_P7 |
| KFD_O1 |
| KFD_Oz |
| KFD_O2 |
| KFD_P4 |
| KFD_P8 |
| KFD_Cz |
| KFD_C4 |
| KFD_T8 |
| KFD_F4 |
| KFD_F8 |
| PEn_Fz |
| PEn_F3 |
| PEn_F7 |
| PEn_C3 |
| PEn_T7 |
| PEn_Pz |
| PEn_P3 |
| PEn_P7 |
| PEn_O1 |
| PEn_Oz |
| PEn_O2 |
| PEn_P4 |
| PEn_P8 |
| PEn_Cz |
| PEn_C4 |
| PEn_T8 |
| PEn_F4 |
| PEn_F8 |
| PFD_Fz |
| PFD_F3 |
| PFD_F7 |
| PFD_C3 |
| PFD_T7 |
| PFD_Pz |
| PFD_P3 |
| PFD_P7 |
| PFD_O1 |
| PFD_Oz |
| PFD_O2 |
| PFD_P4 |
| PFD_P8 |
| PFD_Cz |
| PFD_C4 |
| PFD_T8 |
| PFD_F4 |
| PFD_F8 |
| RR_Fz |
| RR_F3 |
| RR_F7 |
| RR_C3 |
| RR_T7 |
| RR_Pz |
| RR_P3 |
| RR_P7 |
| RR_O1 |
| RR_Oz |
| RR_O2 |
| RR_P4 |
| RR_P8 |
| RR_Cz |
| RR_C4 |
| RR_T8 |
| RR_F4 |
| RR_F8 |
| SFD_Fz |
| SFD_F3 |
| SFD_F7 |
| SFD_C3 |
| SFD_T7 |
| SFD_Pz |
| SFD_P3 |
| SFD_P7 |
| SFD_O1 |
| SFD_Oz |
| SFD_O2 |
| SFD_P4 |
| SFD_P8 |
| SFD_Cz |
| SFD_C4 |
| SFD_T8 |
| SFD_F4 |
| SFD_F8 |
| SVDEn_Fz |
| SVDEn_F3 |
| SVDEn_F7 |
| SVDEn_C3 |
| SVDEn_T7 |
| SVDEn_Pz |
| SVDEn_P3 |
| SVDEn_P7 |
| SVDEn_O1 |
| SVDEn_Oz |
| SVDEn_O2 |
| SVDEn_P4 |
| SVDEn_P8 |
| SVDEn_Cz |
| SVDEn_C4 |
| SVDEn_T8 |
| SVDEn_F4 |
| SVDEn_F8 |
| ShanEn_Fz |
| ShanEn_F3 |
| ShanEn_F7 |
| ShanEn_C3 |
| ShanEn_T7 |
| ShanEn_Pz |
| ShanEn_P3 |
| ShanEn_P7 |
| ShanEn_O1 |
| ShanEn_Oz |
| ShanEn_O2 |
| ShanEn_P4 |
| ShanEn_P8 |
| ShanEn_Cz |
| ShanEn_C4 |
| ShanEn_T8 |
| ShanEn_F4 |
| ShanEn_F8 |
| SpEn_Fz |
| SpEn_F3 |
| SpEn_F7 |
| SpEn_C3 |
| SpEn_T7 |
| SpEn_Pz |
| SpEn_P3 |
| SpEn_P7 |
| SpEn_O1 |
| SpEn_Oz |
| SpEn_O2 |
| SpEn_P4 |
| SpEn_P8 |
| SpEn_Cz |
| SpEn_C4 |
| SpEn_T8 |
| SpEn_F4 |
| SpEn_F8 |
| Theta_Fz |
| Theta_F3 |
| Theta_F7 |
| Theta_C3 |
| Theta_T7 |
| Theta_Pz |
| Theta_P3 |
| Theta_P7 |
| Theta_O1 |
| Theta_Oz |
| Theta_O2 |
| Theta_P4 |
| Theta_P8 |
| Theta_Cz |
| Theta_C4 |
| Theta_T8 |
| Theta_F4 |
| Theta_F8 |
| Theta_ratio_Fz |
| Theta_ratio_F3 |
| Theta_ratio_F7 |
| Theta_ratio_C3 |
| Theta_ratio_T7 |
| Theta_ratio_Pz |
| Theta_ratio_P3 |
| Theta_ratio_P7 |
| Theta_ratio_O1 |
| Theta_ratio_Oz |
| Theta_ratio_O2 |
| Theta_ratio_P4 |
| Theta_ratio_P8 |
| Theta_ratio_Cz |
| Theta_ratio_C4 |
| Theta_ratio_T8 |
| Theta_ratio_F4 |
| Theta_ratio_F8 |
| Theta_watch-relax_Fz |
| Theta_watch-relax_F3 |
| Theta_watch-relax_F7 |
| Theta_watch-relax_C3 |
| Theta_watch-relax_T7 |
| Theta_watch-relax_Pz |
| Theta_watch-relax_P3 |
| Theta_watch-relax_P7 |
| Theta_watch-relax_O1 |
| Theta_watch-relax_Oz |
| Theta_watch-relax_O2 |
| Theta_watch-relax_P4 |
| Theta_watch-relax_P8 |
| Theta_watch-relax_Cz |
| Theta_watch-relax_C4 |
| Theta_watch-relax_T8 |
| Theta_watch-relax_F4 |
| Theta_watch-relax_F8 |
| TotalAbsPow_Fz |
| TotalAbsPow_F3 |
| TotalAbsPow_F7 |
| TotalAbsPow_C3 |
| TotalAbsPow_T7 |
| TotalAbsPow_Pz |
| TotalAbsPow_P3 |
| TotalAbsPow_P7 |
| TotalAbsPow_O1 |
| TotalAbsPow_Oz |
| TotalAbsPow_O2 |
| TotalAbsPow_P4 |
| TotalAbsPow_P8 |
| TotalAbsPow_Cz |
| TotalAbsPow_C4 |
| TotalAbsPow_T8 |
| TotalAbsPow_F4 |
| TotalAbsPow_F8 |
| valence_index |
| arousal_index |
| bpm_watch-relax |
| ibi_watch-relax |
| sdnn_watch-relax |
| sdsd_watch-relax |
| rmssd_watch-relax |
| pnn20_watch-relax |
| pnn50_watch-relax |
| hr_mad_watch-relax |
| sd1_watch-relax |
| sd2_watch-relax |
| s_watch-relax |
| sd1/sd2_watch-relax |
| breathingrate_watch-relax |
| bpm |
| ibi |
| sdnn |
| sdsd |
| rmssd |
| pnn20 |
| pnn50 |
| hr_mad |
| sd1 |
| sd2 |
| s |
| sd1/sd2 |
| breathingrate |
| EDA |
| Corrogator_watch |
| Corrogator_Watch-relax |
| Zygomaticus_watch |
| Zygomaticus_Watch-relax |
